# Medical and Philosophical Intelligence

**Published:** 1813-03

**Authors:** 


### MEDICAL AND PHILOSOPHICAL INTELLIGENCE.

GEOLOGICAL SOCIETY.—At a meeting of the Society on Dec. 4th, a paper, by William Philips, esq. M.G.S. "on the veins of Cornwall," was read; from which it appears that the regular or metalliferous veins of Cornwall are found, with few exceptions, to run east and west. The known length of many of these veins is considerable, amounting, in some instances, to two or more miles; but their actual termination at either extremity has in no case been satisfactorily ascertained, all that is known, being, that they gradually become so poor and narrow as to make it no longer worth the miner's while to pursue them. The dip or descent of the veins varies more or less, from perpendicular inclining towards the north or south, which inclination is called the *underlie* of the lode. The depth of the veins is still less known than their longitudinal extent, not an instance having occurred of a vein being fairly worked out: many mines have indeed been relinquished, but only on account of the expenses of working them exceeding the produce. The deepest mine now in work in Cornwall is Dolcoath, some of the workings of which are 228 fathoms below the surface. The usual width of the veins that are worked, varies from one foot to three; in particular instances, however, portions of veins occur twenty-four feet or even thirty feet wide, and, on the other hand, a vein of tin ore not three inches wide has been followed with profit.

The substances that accompany the metallic ores (or the veinstones) vary considerably, not only in different veins, but in different parts of the same vein; and it is from these, and not from their metallic contents, that the miner's nomenclature of the veins is derived.

Gossan is a friable substance of a loose texture, consisting of clay, mixed more or less with siliceous matter, and coated or tinged with oxide of iron; its color varies from light yellow to deep red and brownish black. A gossany lode is more common than any other, and is considered as promising both for copper and tin.

When quartz predominates, the vein is said to be sparry; and, if the quartz is considerably compact, it is looked upon as a very unfavorable indication, more especially if the vein becomes narrower as it descends.

If iron pyrites abound, the vein is said to be *mundicky*. When this substance occurs at a shallower level, it is considered as not unpromising, more especially if mingled with yellow copper ore as it descends.

A vein containing a large proportion of chlorite is termed a *peachy* lode, and promises for tin rather than for copper.

A vein is said to be *flookany*, when one or both of its sides is lined with blueish white clay. It sometimes is so abundant as to occasion considerable difficulty and expense to prevent it from slipping down and obstructing the works.

When the contents of a vein consist of a hard substance of a greenish or brownish color, which appears to be chiefly a mixture of quartz and and chlorite, the vein is denominated *caply*. Tin is often found in it, copper rarely.

When the ore, whether of tin or copper, is found in detached stones or lumps, mixed loosely with the other contents of the veins, it is termed a *pryany* lode.

A vein abounding in blende is called a *black-jack* lode, and is considered as unpromising for tin, but a good sign of copper.

When a vein contains granite in masses or blocks, or in a state of semi-decomposition, it is termed a *growan* lode, and is generally considered as more promising for tin than for copper. Of late, however, many rich veins of copper have been found in the granite district of Cornwall.

The experienced miner by no means implicitly relies on even the most promising symptoms, for all of them at times are found to mislead; the following, however, are those in favor of which he is more especially prepossessed: all gossany lodes in general, the early discovery of pyrites with portions of yellow copper ore, also of blende and of galena, and the cutting of a good course of water, especially if it be warm.

The discovery of veins is effected in various ways: the ancient mode of *shoding* or tracing up water-courses, when pieces of ore are found to occur among the rolled stones in their channels, is now rarely resorted to. The common method is to work drifts across the country from north to south, by which all veins in the district thus examined are sure to be cut through. Veins are often found in driving adits and levels for the working of known lodes; and not unfrequently are stumbled on by mere accident in digging ditches and foundations of walls.

The contents of a vein may be divided into those which are valuable, and those which are not so; the latter, forming generally by far the largest portion, are technically called *deads*, and are left in the vein both to avoid the unnecessary expense of raising them to the surface, and for the very important purpose of preventing the two walls of the vein from collapsing, and thus destroying the works: in addition to the deads, strong pieces of timber are frequently made use of. Sometimes large wedge-shaped fragments of rock, called by the miners *horses*, occur in the vein, partially cutting off the regular contents of the lode, though seldom, if ever, entirely obstructing it. Veins of copper ore are, however, particularly liable to capricious and total obstructions without any obvious cause. In proportion as the rock becomes harder, the vein always becomes more narrow.

One of the first objects in opening a new mine, is to drive an *adit* or horizontal gallery from the lowest convenient level, for the purpose of carrying off all the top water. One adit often serves two or three mines; and there is one (called the deep adit) which opens on one of the creeks of Falmouth harbour, the entire subterranean length of which is about twenty-four miles.

Copper veins, which fifty years ago were considered by the Cornish miners to be peculiar to schist, have of late been found, in the parishes of Gwennah and Redruth, to pass freely from schist into granite, and and back again to schist, without any deterioration. The texture and hardness of both rocks is liable to considerable variation, affecting, of course, the profit and progress of the miner often in a very remarkable degree. Two shafts of Huel Alfred were sunk in schist, and the cost of the one did not exceed 5*l.* per fathom, while that of the other amounted to 55*l.* for the same length.

The metalliferous, or east and west, veins, are crossed by others, the direction of which is nearly north and south. These latter are called *cross courses,* and rarely produce copper or tin, or any other metallic substances. The principal practical advantage derived from these veins, especially when consisting of clay, is, that they oppose an effectual obstacle to the passage of water, and therefore the miners do not willingly pierce them without some adequate object in view. The disadvantage of them is, that they not only interrupt the course of the metalliferous veins, heaving them from a few inches to several fathoms, but not unfrequently totally impoverish them, so that a long and costly search after the heaved part of a vein often terminates in the mortifying discovery that it is not worth pursuing, as was strikingly exemplified in the corresponding veins of Huel Jewel and Tol Carn.

There is another species of vein called a *contre* or *caunter,* the direction of which is for the most part N.E. and S.W. These are mostly, if not always, metalliferous, and often remarkably rich; of which the mines of Huel Alfred and Herland have afforded most splendid instances.

---

*New Patents.*—Louis Honore Henry Germain Constant, of Bland-ford-street, Portman-square, in the parish of St. Mary-le-bone, and county of Middlesex, for a method of refining sugars. Dated Feb. 27, 1812.—Mr. Constant's method consists in making a very pure syrup, and making it pass slowly through the raw or loaf sugar to be refined. According to him, it drives before it the molasses, or colored syrup, and takes its place. The pure syrup is made by dissolving raw sugar in water, heating the solution, mixing it with five pounds of finely-pounded charcoal for every hundred-weight of sugar, adding the usual proportion of blood, bringing the syrup to boil, stopping the heat by means of a metal plate drawn under the boiler, and then skimming off the albumen and charcoal which collects on the surface. This process, however, is not quite new. Something very similar was proposed by Sir Humphrey Davy, in one of his lectures, several years ago; and, about a year since, Mr. Edward Howard suggested a similar method in the evening paper called the Star, and likewise in the Philosophical Magazine.

Frederic Albert Winsor, of Shooter's-hill, in the county of Kent, esq. for a method of employing raw and refined sugars in the composition of sundry articles of merchandise in great demand, where it has not heretofore been used. Dated Dec. 4, 1811.—Mr. Winsor's proposal is to mix together three parts of gunpowder, and one part of well triturated sugar. The mixture, he says, will have the force of four parts of gunpowder.

*Classification of Diseases, by* David Hosack, M.D. *Professor of the Theory and Practice of Physic and Clinical Medicine in the University of New York.*

### Class. I.—Febres.

#### Order I.—*Intermittentes.*

1. Quotidiana.
2. Tertiana.
3. Quartana.

#### Ord. II.—*Remittentes.*

4. Remittens Biliosa.

#### Ord. III.—*Continuæ.*

5. Synocha.
6. Typhus vel Synochus.
7. Dysenteria Epidemica.
8. Pestis Orientalis.
9. Pestis Occidentalis.

### Class. II.—Phlegmasiæ.

10. Phlogosis.
11. Phrenitis.
12. Ophthalmia.
13. Otitis.
14. Odontitis.
15. Catarrhus.
16. Cynanche Tonsillaris.
17. Cynanche Maligna.
18. Cynanche Trachealis.
19. Cynanche Perstidi.
20. Mastodynia.
21. Pertussis.
22. Pneumonia.
23. Phthisis Pulmonalis.
24. Carditis.
25. Peritonitis.
26. Gastritis.
27. Enteritis.
28. Hepatitis.
29. Splenitis.
30. Nephritis.
31. Cystitis.
32. Urethritis.
33. Hysteritis.
34. Phlegmasia dolens.
35. Rheumatismus.
36. Podagra.
37. Arthropuosis.
38. Hydarthrus.
39. Periostitis.

### Class. III.—Cutanei.*

#### Ord. I.—*Papulæ.*

40. Strophulus.
41. Lichen.
42. Prurigo.

#### Ord. II.—*Squamæ.*

43. Lepra.
44. Psoriosis.
45. Pityriasis.
46. Icthyosis.

#### Ord. III.—*Exanthemata.*

47. Rubeola.
48. Scarlatina.
49. Urticaria.
50. Roseola.
51. Purpura.
52. Erythema.

#### Ord. IV.—*Bullæ.*

53. Erysipelas.

54. Pemphigus.

55. Pompholyx.

#### Ord. V.—*Pustulæ.*

56. Impetigo.

57. Ecthyma.

58. Variola.

59. Scabies.

60. Porrigo.

---

* In this class, Dr. Willan's lucid arrangement of cutaneous diseases is adopted.

Ord.

Ord. VI.—*Vesiculæ.*

61. Herpes.
62. Varicella.
63. Vaccinia.
64. Miliaria.
65. Eczema.
66. Aphthæ.

Ord. VII.—*Tubercula.*

67. Phyma.
68. Verruca.
69. Molluscum.
70. Vitiligo.
71. Acne.
72. Lupus.
73. Elephantiasis.
74. Frambœsia.

Ord. VIII.—*Maculæ.*

75. Ephelis.

76. Nævus.

77. Spilus.

Class. IV.—Profluvia.

Ord. I.—*Hæmorrhagiæ.*

78. Epistaxis.

79. Hæmoptysis.

80. Hæmatemesis.

81. Hepatirrhœa.

82. Hæmaturia.

83. Hæmorrhois.

84. Menorrhagia.

Ord. II.—*Apocenoses.*

85. Ephidrosis.

86. Epiphora.

87. Otirrhœa.

88. Ptyalismus.

89. Galactirrhœa.

90. Cholera.

91. Diarrhœa.

92. Dysenteria.

93. Diabetes.

94. Enuresis.

95. Leucorrhœa.

96. Gonorrhœa.

Class. V.—Suppressiones.

97. Icterus.

98. Obstipatio.

99. Ischuria.

100. Dysuria.

101. Dyspermatismus.

102. Amenorrhœa.

103. Dysmenorrhœa.

104. Suppressio mensium.

105. Dyslochia.

106. Agalactia.

Class. VI.—Neuroses.

Ord. I.—*Paralyses.*

107. Apoplexia.
108. Paralysis.
109. Amaurosis.
110. Dysopia.
111. Pseudoblepsis.
112. Strabismus.
113. Dysecœa.
114. Paracusis.
115. Anosmia.
116. Agheustia.
117. Aphonia.
118. Paraphonia.
119. Psellismus.
120. Dysphagia.
121. Anæsthesia.

Ord. II.—*Adynamiæ.*

122. Asphyxia.
123. Syncope.
124. Dyspepsia.
125. Pyrosis.
126. Bulimia.
127. Satyriasis.
128. Nymphomania.
129. Anaphrodisia.

ORD. III.—*Spasmi.*

a. *In functionibus Animalibus.*

130. Tetanus.

131. Trismus.

132. Dolor faciei.

133. Tremor.

134. Convulsio.

135. Chorea.

136. Epilepsia.

b. *In functionibus Vitalibus.*

137. Palpitatio.

138. Angina Pectoris.

139. Asthma.

c. *In functionibus Naturalibus.*
140. Colica.
141. Nephralgia.
142. Hysteralgia.
143. Hysteria.
144. Hydrophobia.

ORD. IV.—*Vesaniæ.*

145. Amentia.
146. Melancholia.
147. Hypochondriasis.
148. Mania.
149. Oneirodynia.

CLASS. VII.—CACHEXIÆ.

ORD. I.—*Intumescentiæ.*

a. *Adiposæ.*

150. Polysarcia.

b. *Flatuosæ.*
151. Pneumatosis.
152. Tympanites.
153. Physometra.

c. *Aquosæ.*
154. Anasarca.
155. Hydrocephalus.
156. Hydrorachitis.
157. Hydrothorax.
158. Hydrops Pericardii.
159. Ascites.
160. Hydrops Ovarii.
161. Hydrometra.
162. Hydrocele.
163. Hydrops Articuli.

d. *Solidæ.*

164. Physconia.

ORD. II.—*Vitia.*

165. Atrophia.
166. Rachitis.
167. Mollities Ossium.
168. Lithiasis.
169. Scrofula.
170. Syphilis.
171. Sibbens.
172. Scorbutus.
173. Petechiæ sine febre.
174. Plica Polonica.

CLASS. VIII.—LOCALES.

ORD. I.—*Dysæsthesiæ.*

175. Caligo.

ORD. II.—*Tumores.*

176. Aneurisma.
177. Varix.
178. Ecchymoma.
179. Schirrus.
180. Carcinoma.
181. Bronchocele.
182. Sarcoma.
183. Fungus Hæmatodes.
184. Polypus.
185. Lupia.
186. Ganglion.
187. Exostosis.

ORD. III.—*Ectopiæ.*

188. Hernia.
189. Prolapsus.
190. Luxatio.

ORD. IV.—*Dialyses.*

191. Vulnus.
192. Ulcus.

ORD. V.—*Deformitates.*

PRIVATE

### PRIVATE MEDICAL SCHOOL, NEW YORK.

*Plan of Study adopted in the Private Medical School, established by* DAVID HOSACK, M.D. *Professor of the Theory and Practice of Physic and Clinical Medicine, in the University of the State of New York.*

To those Students who have not received a liberal Education, the following Works are recommended previously to the commencement of their Medical Studies.

*Rhetoric and Belles Lettres.*

Murray's Grammar, (large ed.)
Blair's Lectures on Rhetoric,
Duncan's Logic,
Campbell's Philosophy of Rhetoric,
Gregory's Letters on Composition,
Kett's Elements of Human Knowledge,
Barron's Lectures on Belles Lettres and Logic,
Gerard on Genius,
Do. on Taste,
Allison on Taste.

*Geography.*

D'Anville's Ancient Geography,
Pinkerton's Modern do.
Morse's American do.

*History.*

Priestley's Lectures on History,
Tytler's Outlines of General History,
Anquetil's Universal History,
Miller's Retrospect of the 18th Century.

*Natural Philosophy.*

Helsham's Lectures,
Gregory's Economy of Nature,
Cavallo's Natural Philosophy,
Imison's School of Arts,
Ferguson's Astronomy.

*Moral Philosophy.*

Beattie's Elements of Moral Science,
——— Essay on Truth,
Paley's Principles of Moral and Political Philosophy.

It is also recommended to the young gentlemen, unprepared by a collegiate course of study, to attend, at least, one course of lectures on Mathematics and Natural Philosophy, as delivered in Columbia College; and to devote an hour daily to the Latin, Greek, and French, languages, under the direction of a private teacher.

### FIRST YEAR OF MEDICAL STUDIES.

*Anatomy.*

Cheselden's Anatomy,
System of Anatomy from the Encyclopædia Britannica,
Bell's Anatomy,
Fyfe's Anatomy,
Wistar's Anatomy,
Jadelot's Myology, (col. plates,)
Bell, on the Bones, Muscles, &c. (plates,)
——— on the Blood Vessels, (coloured do.)
——— on the Nerves, (do.)
——— on the Brain, (do.)
Cruikshanks on the Absorbents.

*Physiology.*

Haller's Physiology,
Blumenbach's do.
Richerand's do.
Gregory's Conspectus Medicinæ, vol. 1st.

*Chemistry.*

Lavoisier's Chemistry,
Henry's Chemistry,
Black's do.
Jacquin's do.
Murray's Elements of Chemistry.

*Botany.*

Curtis on the Classes and Orders,

Smith's

Smith's Botany,
Miller's Illustrations, (col. plates,)
Willdenow's Botany,
Barton's Elements of Botany.

*Materia Medica.*

Lewis's Dispensatory,
Duncan's Edinburgh Dispensatory,
Murray's System of Materia Medica,
Pearson's Synopsis of do.

*Practice of Physic and Surgery.*

Bell's, B. System of Surgery,
Bell's, C. System of Dissections,
Thomas's Practice of Physic.

SECOND YEAR.

*Anatomy.*

Albinus' Anatomical Plates,
Edinburgh System of Anatomy,
Anatomie de Sabatier,
Cuvier's Comparative Anatomy,
Blumenbach's do.
Baillie's Morbid Anatomy,
Clossy's Dissections of Morbid Anatomy.

*Physiology.*

Moore's Medical Sketches,
Hewson on the Blood,
Falconar and Hewson's Experiments on do.
Hunter on the Blood,
Saumarez's Physiology,
Bostock on Respiration,
Cruikshank on Perspiration,
Physiologie de Dumas,
Fox on the Natural History of the Teeth.

*Surgery.*

Cooper's Surgery,
Bell's, J. Surgery, by Smith
Boyer on the Bones,
Bell's, C. Operative Surgery,
Abernethy's Surgical Observations
Desault's Surgery,
Pott's Surgical Works,
Crowther on White Swelling,
Ford on the Hip Joint,
Cooper on the Joints,
Sheldon on Fracture of the Patella,
Russel on Necrosis,
Fox on the Diseases of the Teeth,
Hunter on the Venereal.

*Chemistry and Mineralogy.*

Murray's System of Chemistry,
Chaptal's Chemistry,
Parkinson's Chemistry,
Accum's do.
Kirwan's Mineralogy,
———— Geology,
Cronstedt's Mineralogy,
Dumeril's Zoology.

*Botany.*

Hull's Botany,
Woodville's Medical Botany, (colored plates,)
Linnæus' Systema Vegetabilium,
———— Genera Plantarum,
Willdenow's Species Plantarum,
Jussieu's Natural Orders,
Persoon's Synopsis.

*Materia Medica.*

Cullen's Materia Medica,
Moore's Strictures on do.
Duncan's Therapeutics,
Gregory's Conspectus Medicinæ, vol. 2d.
Barton's Collections towards a Materia Medica.

*Practice of Physic.*

Cullen's Nosology,
———— Practice of Physic,
Wilson on Febrile Diseases,
Home's Principia Medicinæ,
Saunders on the Liver,
Willich on Diet and Regimen.

*Midwifery.*

Bard's Compendium,
Denman's Midwifery,
Smellie's Plates,
Hunter on the Gravid Uterus, (plates.)

THIRD YEAR.

*Anatomy.*

Vicq D'Azyr, on the Brain, (colored plates,)

Scarpa Tabulæ Nervorum, (plates)

Monroe on the Brain, Eye, and Ear,

———— on the Nervous System, (plates,)

Sheldon

Sheldon on the Absorbents,
Morgagni on the Seat and Causes of Disease.

*Practice of Physic.*

Fordyce on Fevers,
Huxham on Fevers,
Pringle on Diseases of the Army,
Blane's Diseases of Seamen,
Cleghorn's Diseases of Minorca,
Lind's Works,
Rush's Works,
Percival's Essays,
Fothergill's Works,
Reid on Consumption,
Whytt on Nervous Diseases,
Moseley on Tropical Diseases,
Senac on Fevers,
Heberden's Commentaries,
Duncan's Medical Cases,
Chisholm on the Malignant Fever,
Currie's Medical Reports,
Saunders on Mineral Waters,
Dickson on Gravel and Gout,
Wilson on Dyspepsia,
Young on Opium,
Crumpe on do.
Matthias on the Mercurial Disease
Swediaur on the Venereal,
Bell on do.
Pearson on do.
M'Bride's Practice of Physic,
———— Experiments,
Trotter on the Nervous Temperament,
Warren on Diseases of the Heart,
Corvissart on do.
Willan on Diseases of the Skin, (colored plates,)
Ferriar's Medical Histories and Reflections,
Robertson's History of the Atmosphere,
Hufeland on Long Life,
Alexander's Experiments,
Hamilton on Purgatives,
Richter's Observations,
Cogan on the Passions,
Pemberton on the Abdominal Viscera,
Sydenham's Works,
Boerhaave's Aphorisms,
Van Swieten's Commentaries,
Hoffman's Practice,
Celsus de Medicina.

*Surgery.*

Bell, J. Surgery, (plates,)
Chirurgie de Pouteau,
Cooper on Hernia, (plates,)
Camper on Hernia, (plates,)
Scarpa on Aneurism,
——— on the Eye,
Saunders on the Ear, (plates,)
Ware on the Eye, Hæmorrhoids, &c.
Hey's Practical Observations on Surgery,
Jones on Hæmorrhage.

*Chemistry.*

Thomson's System of Chemistry,
Johnson's Animal Chemistry.

*Materia Medica.*

Alston's Materia Medica,
Murray's Apparatus Medicaminum.

*Midwifery, and the Diseases of Women and Children.*

Hamilton, A. Works on Midwifery
———— J. jun. do.
Denman's Aphorisms,
Clark's Midwifery,
Burn's Works on Midwifery,
Baudeloque's Midwifery,
White on Lying-in Women,
White on the Swelling of the Lower Extremities of Lying-in Women,
Perfect's Cases of Midwifery,
Cheyne's Diseases of Children,
Underwood's do.
Heberden's do.
Sparman's do.
Cheyne's Pathology of the Larynx and Trachea.

*Physiology and Pathology of the Human Mind.*

Reid's Inquiry,
——— Active Powers,
——— Intellectual Powers,
Hartley on Man,
Stewart's Philosophy of the Human Mind,
———— Philosophical Essays,

Crighton

Crighton on Mental Derangement
Arnold on Insanity,
Haslam on Madness,
Pinel on Insanity.

*History of Medicine.*

Cabanis' Revolutions in Medicine
Friend's History of Medicine,
Le Clerc's do.
Hilary on the Means of Improving Medical Knowledge,
Percival's Medical Ethics,
Zimmerman on Experience.

*Books of Reference.*

Martyn's Language of Botany,
Milne's Botanical Dictionary,
Turton's Medical Glossary,
Cooper's Surgical Dictionary, by Dorsey,
Nicholson's Chemical Dictionary,
Aikin, J. and C. do.

*Periodical Works, recommended to be read.*

London Medical Transactions,
London Medical Observations and Inquiries,
Physical and Literary Essays of Edinburgh,
Chirurgical Transactions, by Hunter, Home, &c.
London Medical Museum,
London Medical Communications
Memoirs of the Medical Society of London,
Medical Facts and Observations,
Duncan's Medical Commentaries,
———— Annals of Medicine,
Edinburgh Medical and Surgical Journal,
Medical and Chirurgical Review,
London Medical Review,
Annual Medical Register and Review,
Transactions of the Medical Society of London,
Memoires de L'Academie Royale de Chirurgie,
Journal de Medicine,
Bibliotheque Medicale,
Nicholson's Philosophical Journal,
Tilloch's Philosophical Magazine,
Annals of Philosophy,
Transactions of the Royal Society of Edinburgh,
Memoirs of the Manchester Society,
Transactions of the American Philosophical Society,
Memoirs of the American Academy of Arts and Sciences,
Memoirs of the Connecticut Academy of Arts and Sciences,
Transactions of the College of Physicians of Philadelphia,
New-York Medical Repository,
Coxe's Philadelphia Medical Museum,
Barton's Medical and Physical Journal,
Massachusetts Medical Communications,
New-York Philosophical Journal and Review,
Eclectic Repository,
American Medical and Philosophical Register,
Potter's Medical Lyceum,
New-England Journal of Medicine and Surgery.

In the preceding list are enumerated those writings on medicine and the auxiliary branches of science, an acquaintance with which is deemed essential to constitute the basis of a medical education. And, though it is not expected that any student, however laborious, can render himself familiar with them during the short period of time to which his studies are necessarily limited, yet it is to be hoped, that thus seeing a plan of study before him, he will remit no honorable exertion to its successful acquisition. The works of those distinguished writers will have taught him duly to appreciate the dignity and importance of his profession, and in the intervals of practice he will be induced to complete whatever of this outline he shall have left imperfect. The private library of Dr. Hosack, consisting of the most

 valuable valuable medical writings, ancient and modern, is also constantly open to the student for the sake of reference and more satisfactory information. In addition to this source of instruction, he is also supplied with anatomical preparations and other apparatus necessary to make him acquainted with the structure and the diseases of the human frame. And, for the more complete accomplishment of his plan, Dr. Hosack has associated himself with John W. Francis, M.D. by whose assistance he is enabled to afford to his pupils the advantages of daily lectures and examinations throughout the year.

Suitable apartments are also provided for the accommodation of the student, and such regulations adopted as will secure the benefits both of a private and a public system of instruction.

---

*Deaths* by Small-pox, from the Bills of Mortality, since the last Report.*

| | | | | |
|---|---|---|---|---|
| | Dec. 8, | 72 | | |
| | 15, | 55 | | |
| | Total of Deaths within the year to this day, to which period the yearly bill was made up | | | 1287 |
| | Died of Small-pox in the preceding year | | | 761 |
| | Increase | | | 526 |

*From the Weekly Bills since.*

| | | | | |
|---|---|---|---|---|
| | Dec. 22, | 36 | | |
| | 29, | 35 | | |
| 1813. | Jan. 5, | 31 | | |
| | 12, | 29 | — | 131 |
| | 19, | 25 | | |
| | 26, | 18 | | |
| | Feb. 2, | 41 | | |
| | 9, | 33 | — | 117 |
| | In two months | | | 248 |

---

*Royal College of Surgeons.*—Lectures, 1813.—The first part of the Course of Lectures, of the present year, on Comparative Anatomy, will commence on Tuesday the 16th day of March next, at three in the afternoon, and be continued on Tuesdays and Thursdays until completed.

---

* This would be more properly termed *Burials*; for, although all *Deaths*, within the bills, are reported by the searchers to the parish-clerks, the burials alone at the church are returned by the latter to the Company's Hall.

When, therefore, it is recollected, that a large proportion of those who die are interred in the burial-grounds of dissenters, the actual mortality must obviously be considerably (perhaps, at least, one-third) greater, than appears upon the face of the weekly bills. An inaccuracy which is increased by the total absence of returns from the very extensive and populous parishes of St. Pancras and Mary-le-bonne, which are not comprehended within the Bills of Mortality. In the latter parish, indeed, death makes its way, unnoticed even by the feeble inquiries of a parish searcher. C. M.

The second part of the course, on Human Anatomy and Diseases, will begin on Tuesday, the 12th day of October next.

Tickets for the admission to this Course of Lectures, of Members, and, also, of Pupils, on presenting tickets of attendance on the chirurgical practice of the hospitals in London, will be delivered at the College, between the hours of twelve and three, during the first week in March.

---

Dr. Squire will, on Saturday the 20th of March, begin a Course of Lectures on the Theory and Practice of Midwifery, and the Diseases of Women and Children.

---

The General Committee of Apothecaries have published an "Abstract of a Bill for regulating the Practice of Apothecaries, Surgeon-Apothecaries, and Practitioners in Midwifery, and Compounders and Dispensers of Medicine, throughout England and Wales." As a document of great importance, not only to the medical profession, but to the public at large, we should have been glad to have laid it verbatim before our readers this month, but it came to hand too late. In our next number, however, it will certainly be inserted; and for the present we have only to remark, that both in principle and detail it seems calculated to do great service to the community in general, if passed into a law.

*Subscriptions received from the Country.*

(Continued from p. 172.)

DEVONSHIRE.

*County Meeting, at Exeter.*

J W. Abbot, esq. in the Chair.
Abbott, J. W. Chairman
Arscott, Teignmouth
Adams, Salterton
Anthony, Thoverton
Bartlett, I. B. Teignmouth
Bartlett, I. S. Torquay
Bowden, H. Bradninch
Bowden, H. S. ditto
Butler, Woodbury
Browne, St. Mary Church
Black, Exmouth
Cartwright, Teignmouth
Croker, Bovey Tracey
Collins, Kenton
Cawley, Topsham
Day, Chudleigh
Gater, Exeter
Gaye, Newton Abbot
Gabriel, Cullompton
Goss, Dawlish
Harris, Exeter
Hodge, Honiton Clyst
Hugo, T. Crediton
Hugo, S. ditto
Hawkes, Okehampton
Hole, Morchard Bishop
Howell, Renton
Houston, Thoverton
Holman, W. Crediton
Holman, W. H. ditto
Hurley, Cullumpton
Johnson, Exeter
Kiernan, Broadhempstone
Kingdon, ditto
Lethbridge
Langdon, W. Bampton
Langdon, T. ditto
Lang, Ipplepen
Nosworthy, Crediton
Pridham, Topsham
Pridham, Lympstone
Puddicombe, Moreton
Rowe, Exmouth
Radford, P. Exeter
Radford, B. T. Chumleigh
Smeedon, Chumleigh
Sowden, Exeter
Snelling, J. G. Exeter
Snelling, G. G. ditto
Spry, Silverton
Stocker, Sidmouth
Salter, Clyst Hydon
Sweet, Exeter
Tuke, Dawlish
Thomson, Exeter
Yard, Topsham

Plymouth.

*Plymouth.*

By I. H. Fuge, esq.

Bone, Plymouth Dock
Dunning, ditto
Fuge, I. H. Plymouth
Fuge, T. ditto
Kerswill, Plymouth Dock
Lowe, ditto
Lockyer, Dr. Plymouth
Leecombe, ditto
May, Dr. ditto
Stewart, Dr. ditto
Tenthwill, Plymouth Dock
Williams, Plympton.

*Modbury.*

Gaye, Newton Abbott
Hunt, Dartmouth
Searle, Kingsbridge.

*Axminster District.*

By Henry Corbin, esq.

Arnold, Axminster
Corbin, Colyton
Hayman, Axminster
Jolliffe, Crewkerne
Phillips, Chard
Symes, Axminster
Spicer, Chard
Wright, Seaton.

SOMERSETSHIRE.

*County Meeting, at Wells.*

I. Bond, esq. in the Chair.

Bernard, Wookey
Bryer, Somerton
Bond, Glastonbury
Chaffey, Murtock
Fussel, Temple
Frond, Frome
Goldesborough, Banwell
George, Burton
Glanville, Wedmore
Good, Axbridge
Hamlyn, Murtock
Harding, Cross
Lee, Wells
Miller, Frome
Mansford, ditto
Merling, Bethel
Napper, Frome
Nicholls, Wells
Phippes, Wedmore
Parsley, Banwell
Spencer, Wells
Sweeting, ditto
Strong, North Petherton
Sherland and Moore, Yeovil
Uphill, Somerton
Westcote, Murtock
Warren, Melverton.

*Wellington District.*

King, Bath
Leroux, Clifton
Surrage, Wincanton.

*Bristol.*

I. Paine Berjeu, esq. in the Chair.

Resolutions received, but not the Names of the Subscribers.

STAFFORDSHIRE.

*County Meeting at Stafford.*

F. Hughes, esq. in the Chair.

Resolutions received, but not the Names of the Subscribers.

*Walsall.*

By T. Adams, esq.

Adams, Walsall
Elwell, ditto
Parnell, ditto
Pitt, ditto
Woollatt, ditto
Weaver, ditto

Coombe, Newcastle

Forster, Stone

Proud, Wolverhampton.

YORKSHIRE.

*Halifax.*

G. Alexander, esq. in the Chair.

Alexander, Halifax
Drake, ditto
Dynely, Hebden Bridge
Heyworth, Todmorden
Hardman, ditto
Howard, Heptenstall
Lightfoot, Halifax
Morley, ditto
Shaw, ditto
Thomas, Hebden Bridge

Harrison, Kirky Moorside

Ness, Helmsley

Sandwith, ditto

Wilson, Hovingham

Appleton, Stokesley
Birdsell, Pickering.

(To be continued.)

BOTANICAL

